# Awareness and best practices in using ketogenic therapy to treat serious mental illness: a modified Delphi consensus

**DOI:** 10.3389/fnut.2026.1749406

**Published:** 2026-02-24

**Authors:** Georgia Ede, Matthew Bernstein, Lori Calabrese, Iain H. Campbell, Nicole Laurent, Chris M. Palmer, Shebani Sethi, Beth Zupec-Kania

**Affiliations:** 1 Georgia Ede MD PLLC, Newburyport, MA, United States; 2 Medical Director, Ellenhorn LLC and Accord Mental Health LLC, Arlington, MA, United States; 3 Touchpoints180, South Windsor, CT, United States; 4 Division of Psychiatry, Center for Clinical Brain Sciences, University of Edinburgh, Kennedy Tower, Royal Edinburgh Hospital, Edinburgh, United Kingdom; 5 Licensed Mental Health Counselor, Family Renewal Inc, Vancouver, WA, United States; 6 Harvard Medical School, McLean Hospital, Belmont, MA, United States; 7 Department of Psychiatry and Behavioral Sciences, Stanford University, Stanford, CA, United States; 8 Ketogenic Therapies LLC, Elm Grove, WI, United States

**Keywords:** bipolar disorder, ketogenic diet, major depressive disorder, schizophrenia, serious mental illness

## Abstract

**Background:**

Metabolic dysfunction is emerging as an important contributor to the pathophysiology of major depressive disorder, bipolar disorder, and schizophrenia, fueling interest in ketogenic metabolic therapy (KMT) as a potentially beneficial intervention for serious mental illness. KMT has been used successfully for decades in treating epilepsy, but evidence for treating mental illness has yet to mature.

**Aims:**

This study aimed to produce expert-informed guidance for the implementation of KMT in adults with serious mood and psychotic disorders.

**Method:**

A modified Delphi methodology was used to examine the opinions of KMT-experienced mental health experts. A steering group of eight such experts convened to develop an online survey comprising 33 statements regarding 1) the definition of KMT in the context of serious mood and psychotic illness; 2) identification of eligible candidates; 3) monitoring and measurement standards; and 4) best practices in employing KMT. This survey was distributed to clinician peers to examine opinions. The threshold for consensus agreement was set *a priori* at 75%.

**Result:**

Consensus was reached for all 33 statements (100%); therefore, the steering group approved the complete series of recommendations.

**Conclusions:**

This consensus provides expert-informed guidance to support the use of KMT in adults with major depressive disorder, bipolar disorder, and schizophrenia.

## 1 Introduction

Schizophrenia, bipolar disorder, and major depressive disorder afflict hundreds of millions of people globally (1) and are associated with poor quality of life, reduced life expectancy, and high socioeconomic burden (2).

Despite decades of prescription drug treatments designed to remediate underlying dysfunction in neurotransmitter systems, this approach continues to leave many individuals without meaningful relief (3). Those who do benefit from psychotropic drugs often suffer serious side effects that negatively impact quality of life, cause metabolic dysfunction, and can even be life-threatening (1, 2, 4, 5). Pharmaceutical innovation in psychotropic medication has essentially stalled, with few drugs with novel mechanisms of action approved in recent years (6). This lack of new and effective treatment options is particularly challenging for individuals whose conditions have not responded to multiple psychotropic drugs and are therefore considered treatment-resistant (3, 7–10).

For these reasons, the search for modifiable factors underlying serious mental illnesses has expanded beyond neurotransmitter system dysfunction to include neuroinflammation and excessive oxidative stress (both of which disrupt neurotransmitter production pathways), and insulin resistance—a common driver and marker of general metabolic dysfunction (11, 12). Serious mood and psychotic disorders are strongly correlated with metabolic disorders (13, 14). For example, people with glucose levels in the prediabetes range are 2.7 times more likely to develop major depression (15), those with newly diagnosed bipolar disorder are 3.5 times more likely to have metabolic syndrome (16); and individuals with newly diagnosed schizophrenia are 3.7 times more likely to have insulin resistance (17, 18). Insulin resistance is linked to cerebral glucose hypometabolism, which has been observed in schizophrenia, bipolar disorder, and treatment-resistant major depressive disorder (19). It has been demonstrated in a carefully controlled clinical trial that reversing insulin resistance with medication (metformin) may substantially improve depression symptoms in adults with treatment-resistant bipolar disorder (20), suggesting that addressing metabolic dysfunction by other means such as with lifestyle changes may also offer clinical benefits.

Ketogenic metabolic therapy (KMT), an intervention that leads to potentially therapeutic levels of ketone bodies in the blood, can improve metabolic health (18). While it is possible to lower insulin levels enough to initiate ketogenesis through fasting or significant caloric restriction (21), or raise circulating ketone levels using exogenous ketone (beta-hydroxybutyrate) supplements (11), the safest way to maintain ketosis long-term is with a ketogenic diet.

KMT has been shown to decrease neuroinflammation, regulate neurotransmitter systems, reduce hyperexcitability of the neural network, stabilize neuronal firing rates, and improve brain energy metabolism (11). Indeed, mitochondrial dysfunction is increasingly implicated in the pathogenesis of many neuropsychiatric disorders (22) including schizophrenia (23), bipolar disorder (24), and major depressive disorder (25). KMT improves brain bioenergetics by bolstering antioxidant defenses, which protects the health of existing mitochondria, and by promoting mitochondrial biogenesis, the creation of new mitochondria (26).

The clinical utility of KMT has been demonstrated in epilepsy for many decades (27), and a growing body of evidence that includes preclinical research and human case reports suggests a similar approach could improve both metabolic dysfunction and serious psychiatric symptoms, even supporting remission of treatment-resistant mood and psychotic illnesses in some cases (28, 29).

Although data from larger, more rigorous human clinical trials of KMT in serious mood and psychotic illnesses are not yet available, a growing number of clinicians have been turning to KMT in an effort to improve outcomes for patients living with these challenging conditions.

Our objective was to engage KMT-experienced mental health experts in a modified Delphi study to develop clear clinical guidance for the safe and appropriate implementation of KMT in the treatment of serious mood and psychotic illnesses.

## 2 Materials and methods

This study did not require registration because neither the assigned interventions nor the outcomes assessed were related to the health of participants.

**Figure 1:**
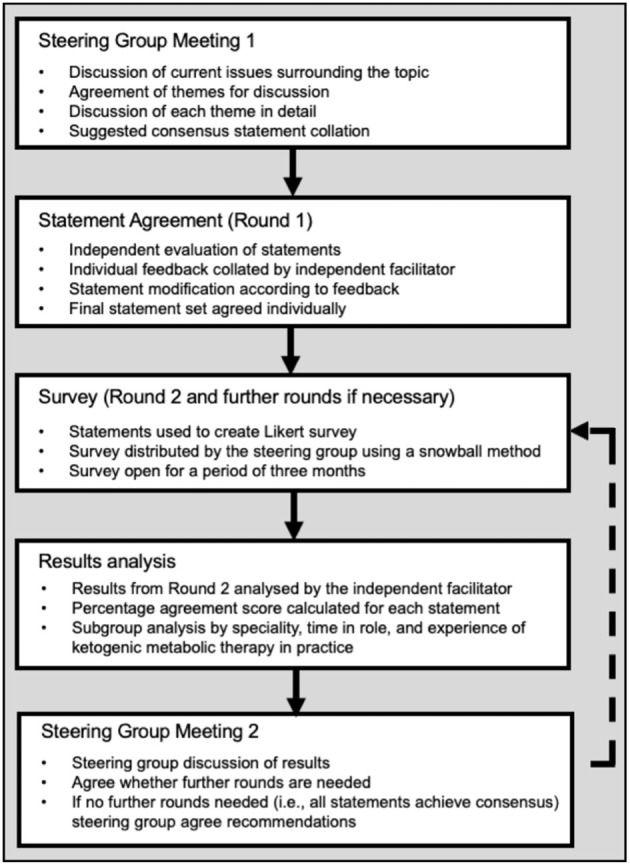
Modified Delphi study stages and objectives.

The process followed a modified Delphi methodology (30) (Figure 1), a well-established approach to answering a research question through identification of a consensus view across subject experts (31). We developed statements which were subsequently circulated to an anonymous wider panel of stakeholders to consolidate current best practice into expert recommendations.

### 2.1 Literature review

We reviewed the literature on the topic of ketogenic therapy in mental illness in October 2023, using PubMed, Google Scholar, and clinical trial registration databases. Search terms included but were not limited to: “ketogenic therapy”, “ketogenic diet”, “ketosis”, “bipolar disorder”, “schizophrenia”, and “major depressive disorder”, and this was supplemented by a general web search using free text terms.

### 2.2 Steering group

We convened a steering group in November 2023 to discuss the principles and practicalities of using KMT in serious mood and psychotic disorders. The group comprised 8 mental health experts (5 clinical psychiatrists, 1 licensed mental health counselor, and 1 registered dietitian all based in the United States, and 1 metabolic psychiatry research fellow based in Scotland) and was guided by an independent facilitator (Triducive Partners). Selected panelists represented leaders in their respective core disciplines (psychiatry, nutrition, behavioral health, and clinical research) who had demonstrated a considerable degree of clinical, scholarly, and/or research experience specifically pertaining to the use of KMT in adults with bipolar disorder, schizophrenia, and/or major depressive disorder.

We developed key questions based on the literature review to drive the meeting discussion. The meeting focused on four broad topics:

A The definition of KMT in the context of serious mood and psychotic disorders
B Identification of optimal candidates for KMT
C Measurement and monitoring standards for KMT
D Best practices in employing KMT

Round 1: Working collaboratively, the group discussed each topic in turn and suggested 31 statements. The group then independently rated the statements as either “accept”, “remove”, “reword”, or suggested additional statements. Recommendations were accepted based on a simple majority.

Round 2: The resulting statements were developed into a *Likert* survey for distribution to a wider panel of peers in the group's professional networks by email using a snowball method. Panelists were experienced mental health experts with knowledge of the use of KMT in practice. Round 2 responses were collected and aggregated by an independent facilitator (Triducive Partners). Although the survey was sent to known individuals to ensure appropriate expertise, responses were anonymized.

Stopping criteria were 1) a 3-month window to collect responses; 2) a target of 50 responses; and 3) 90% of statements passing the (widely accepted) consensus threshold of 75% (32).

A statement of consent was included at the start of the survey, and consent was implied by completion and submission of the survey. As this study only collected the anonymous opinions of mental health professionals, and no identifying or patient-specific data was captured, ethical approval was not required.

Responses were captured using Microsoft Forms technology by the independent facilitator. Panelists were not compensated.

Completed surveys were analyzed in April 2024 using Microsoft Excel software. Responses were aggregated to provide an overall agreement level (i.e., the number of respondents expressing agreement as a percentage of the overall number of responses for each statement). Two weeks later, the steering group reviewed the responses to determine how they guided the conclusions and recommendations to be put forward.

### 2.3 Patient and public involvement

None. The stated objective was to examine the opinions of mental health experts regarding the principles of KMT use in serious mood and psychotic disorders and provide clinical guidance for implementation.

## 3 Results

During Round 1, of the initial 31 statements, 5 were removed, 20 were reworded, and 7 new statements were added, resulting in a final agreed set of 33 statements (S1–S33).

At the end of Round 2, 99 respondents initiated the survey, 52 of whom did not meet the screening criteria (i.e., did not currently work in the mental health field, reported no knowledge of ketogenic or metabolic therapies, or were not using ketogenic metabolic therapies in practice). The remaining 47 respondents completed the survey and were included in the analysis of results (Table 1). Respondents' roles included physician (*n* = 24), psychologist (*n* = 1), dietitian (*n* = 8), health coach (*n* = 6), therapist (*n* = 5), nurse (*n* = 3), or a combination of these. The majority of respondents (37/47) had greater than 5 years of experience in their role, 13/47 respondents described their knowledge of ketogenic and other metabolic therapies as “very experienced/expert”, 27/47 as “some experience in practice”, and 7/47 as having awareness but not experienced in practice.

**Table 1:** Defined consensus statements and corresponding levels of agreement.

**No**.	**Statement**	**Strongly agree**	**Tend to agree**	**Tend to disagree**	**Strongly disagree**	**Agreement**
**Topic A: the definition of ketogenic therapy in the context of serious mental illness** ^*^						
S1	Ketogenic metabolic therapy (historically known as ketogenic diet) is an established evidence-based treatment used for over 100 years to treat epilepsy	85.1%	12.8%	2.1%	0.0%	97.9%
S2	Ketogenic metabolic therapy is supported by 6 RCTs in adults with epilepsy and numerous scientific articles describing a variety of mechanisms of action	72.3%	27.7%	0.0%	0.0%	100.0%
S3	Ketogenic metabolic therapy shows promise in treating serious mental illness	83.0%	17.0%	0.0%	0.0%	100.0%
S4	Ketogenic metabolic therapy involves the modification of food intake that results in the production of endogenous ketone bodies and a state of nutritional ketosis	83.0%	17.0%	0.0%	0.0%	100.0%
S5	Ketogenic metabolic therapy requires a minimum elevation in serum ketones, typically 0.5 mmol/L, for a substantial duration (≥3 months) for measurable benefits	31.9%	57.4%	8.5%	2.1%	89.4%
**Topic B: the optimal candidate for ketogenic therapy**						
S6	Individuals with serious mental illness are suitable candidates for ketogenic metabolic therapy, especially if they show peripheral signs of metabolic dysfunction	46.8%	44.7%	6.4%	2.1%	91.5%
S7	Individuals with serious mental illness without peripheral signs of metabolic dysfunction may benefit from ketogenic metabolic therapy, as they may have metabolic dysfunction in their brain in the absence of peripheral signs	38.3%	51.1%	10.6%	0.0%	89.4%
S8	Individuals without absolute contraindications are suitable candidates for ketogenic metabolic therapy	68.1%	29.8%	2.1%	0.0%	97.9%
S9	Ketogenic metabolic therapy may not be suitable for some individuals living with a serious mental illness	66.0%	31.9%	2.1%	0.0%	97.9%
S10	Motivated individuals and those with at least one support person are most likely to be suitable candidates for ketogenic metabolic therapy	59.6%	29.8%	4.3%	6.4%	89.4%
S11	Individuals without significant motivation and/or personal support may also succeed with ketogenic metabolic therapy in some situations, for example, in an inpatient or residential setting	68.1%	31.9%	0.0%	0.0%	100.0%
S12	Individuals who are unable to tolerate treatment side-effects are suitable candidates for ketogenic metabolic therapy	53.2%	40.4%	6.4%	0.0%	93.6%
S13	Those who decline pharmaceutical treatments are suitable candidates for ketogenic metabolic therapy	40.4%	57.4%	2.1%	0.0%	97.9%
S14	Individuals with treatment refractory disease are suitable candidates for ketogenic metabolic therapy	57.4%	40.4%	2.1%	0.0%	97.9%
S15	Individuals should be offered ketogenic metabolic therapy as an adjunct to first-line treatment	31.9%	63.8%	4.3%	0.0%	95.7%
S16	All suitable patients with serious mental illness should be offered a trial of ketogenic therapy if they wish	36.2%	61.7%	2.1%	0.0%	97.9%
**Topic C: the monitoring and measurement standards for ketogenic therapy**						
S17	Clinically relevant ketosis typically starts at a serum β -hydroxybutyrate (BHB) concentration of 0.5 mmol/L	34.0%	66.0%	0.0%	0.0%	100.0%
S18	In some patients using ketogenic metabolic therapy, the greatest improvements in psychiatric symptoms are observed when serum ketone levels [i.e., β -hydroxybutyrate (BHB)] are consistently maintained around 1.0 mmol/L or higher	46.8%	48.9%	2.1%	2.1%	95.7%
S19	Consistent elevation of serum ketones (at least 0.5 mmol/L) for an extended duration of 6-12 weeks of ketogenic metabolic therapy may be required for observable improvements in serious mental illness	59.6%	38.3%	2.1%	0.0%	97.9%
S20	The duration for ketogenic metabolic therapy to realize observable benefits is highly variable and may take 3-4 months	42.6%	53.2%	4.3%	0.0%	95.7%
S21	During the initiation phase of ketogenic metabolic therapy (≤ 3 months), serum β-hydroxybutyrate (BHB) should be monitored regularly	78.7%	21.3%	0.0%	0.0%	100.0%
S22	Alternative methods of measuring β-hydroxybutyrate (BHB) such as breath or urine should be considered if serum measures are unavailable or impractical.	48.9%	46.8%	4.3%	0.0%	95.7%
S23	Prior to starting ketogenic metabolic therapy, baseline complete blood count, comprehensive metabolic panel, fasting lipid profile, fasting insulin, vitamin B12, vitamin D level, and a carnitine panel are recommended	46.8%	46.8%	6.4%	0.0%	93.6%
S24	Regular monitoring of complete blood count, comprehensive metabolic panel, fasting lipid profile, fasting insulin, vitamin B12, vitamin D, and carnitine is recommended for individuals using ketogenic metabolic therapy	78.7%	21.3%	0.0%	0.0%	100.0%
S25	In individuals using ketogenic metabolic therapy, deficiencies in nutrients such as vitamin D, vitamin B12 and L-carnitine should be addressed with supplementation	63.8%	34.0%	2.1%	0.0%	97.9%
S26	Adequate hydration and electrolyte intake is important during the initiation and maintenance of ketogenic metabolic therapy	53.2%	44.7%	2.1%	0.0%	97.9%
**Topic D: the best practices in employing ketogenic therapy**						
S27	Ketogenic metabolic therapy should be personalized to the needs and preferences of the patient	70.2%	27.7%	2.1%	0.0%	97.9%
S28	Individuals using ketogenic metabolic therapy to treat serious mental illness should be supported by a dietitian/licensed nutritionist or health coach trained in ketogenic metabolic therapy and a prescribing clinician, or a prescribing clinician with specific training in ketogenic metabolic therapy	61.7%	29.8%	8.5%	0.0%	91.5%
S29	Regular monitoring of metabolic markers such as weight, BMI, and waist circumference is recommended	36.2%	59.6%	4.3%	0.0%	95.7%
S30	Individuals should be monitored for the emergence or worsening of symptoms of serious mental illness, which can occur more frequently during the first few weeks of treatment	34.0%	57.4%	8.5%	0.0%	91.5%
S31	Psychiatric medication adjustments may be required during ketogenic metabolic therapy and should be managed by a prescribing clinician	76.6%	23.4%	0.0%	0.0%	100.0%
S32	Blood sugar or blood pressure regulation medications, including diuretics and carbonic anhydrase inhibitors, often require frequent and potentially early adjustments during ketogenic metabolic therapy and should be managed by a prescribing clinician	74.5%	23.4%	2.1%	0.0%	97.9%
S33	Education in the use of ketogenic metabolic therapy for psychiatrists, dietitians, nutritionists and health coaches should be a priority for the mental health field	74.5%	21.3%	4.3%	0.0%	95.7%

^*^schizophrenia, bipolar disorder or major depressive disorder. RCT = randomized controlled trial; BMI = body mass index.

As the stopping criteria were not satisfied, the steering group agreed to extend the window for collection for an additional month with the aim of achieving 50 responses. One appropriate additional response was received (47 responses total).

**Figure 2:**
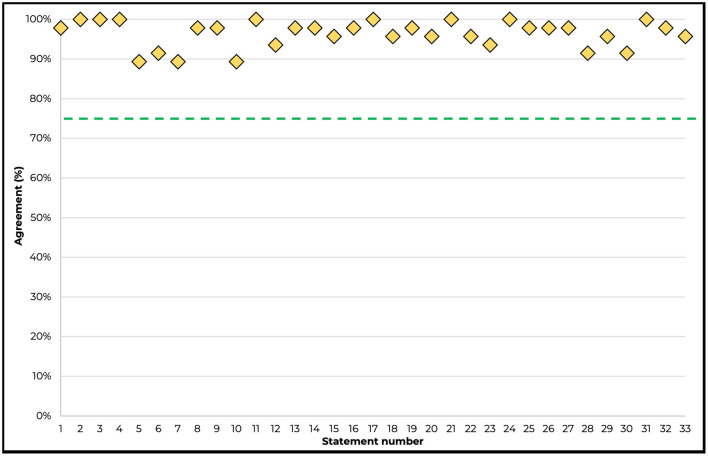
Consensus agreement levels by statement. The threshold for consensus is depicted by the green line (75%).

All statements achieved consensus agreement, with >91% of respondents strongly agreeing on 30/33 (91%) statements, and between 75% and 91% tending to agree on 3/33 (9%) statements (Table 1, Figure 2).

**Figure 3:**
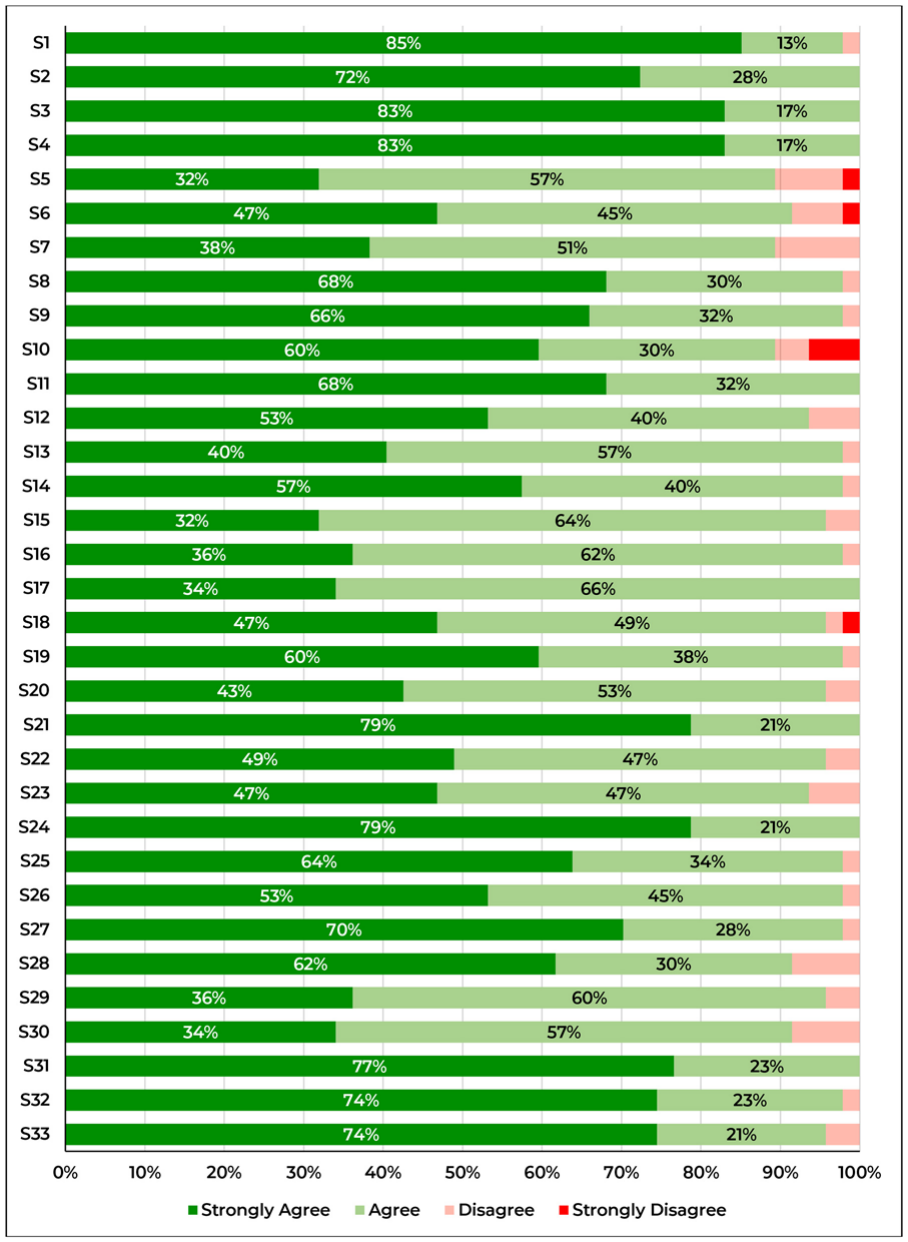
Percentages of agreement level by statement.

Distribution of consensus scores on the 4-point Likert scale provided to respondents is represented in Figure 3.

## 4 Discussion

Panelists reached consensus across all 33 statements. The steering group then used these statements to create the following recommendations, which can be used to inform key considerations for clinicians wishing to employ KMT in the treatment of major depressive disorder, bipolar disorder, and schizophrenia.

### 4.1 Topics

#### 4.1.1 Topic A: The definition of ketogenic therapy in the context of serious mental illness

Respondents agreed that the ketogenic metabolic state should ideally be achieved through modifications of food intake that support the production of endogenous ketone bodies (S4, 100%), specifically by transitioning to a very-low-carbohydrate, adequate-protein, high-fat ketogenic diet. Ketogenic diets should be constructed to provide all essential nutrients, and the proteins and fats they contain may be animal-sourced and/or plant-sourced (33).

Respondents were aware of Level 1 evidence supporting the use of KMT in epilepsy (S1, 98%; S2, 100%) and agreed that there is clear potential for use in serious mental illness (S3, 100%). Respondents agreed that clinically relevant ketosis begins at 0.5 mmol/L beta-hydroxybutyrate (BHB), but that many individuals may require BHB levels of 1.0 mmol/L or higher for best outcomes (S5, 89%). Clinical response times are highly variable; respondents agreed that it may take several weeks to several months of consistent ketosis for measurable clinical benefits of KMT to manifest (S5, 89%; S19, 98%).

#### 4.1.2 Topic B: The optimal candidate for ketogenic therapy

This topic was intended to identify adults with major depressive disorder, bipolar disorder, and schizophrenia who are most likely to benefit from KMT. Respondents agreed that a trial of KMT is appropriate for most adults with these serious mood and psychotic illnesses (S6, 91%; S7, 89%). However, there are a few absolute contraindications to be aware of that make proceeding with KMT potentially dangerous, and many relative contraindications that warrant careful consideration before deciding whether to initiate KMT (S9, 98%). For these reasons, a detailed medical and psychiatric history must always be taken prior to considering KMT.

Table 2 lists medical conditions viewed as absolute and relative contraindications to KMT according to three recent authoritative reviews (34–36). Expert opinions vary, most notably with respect to severe hypertriglyceridemia and hypercholesterolemia, which are viewed as *absolute* contraindications by the National Lipid Association (34) but as *relative* contraindications by other sources. Although there is mounting evidence that elevated LDL cholesterol does not pose a direct risk to cardiovascular health (37–40), the authors recommend caution in these cases, as the elevations in LDL cholesterol that sometimes accompany carbohydrate restriction could exacerbate pre-existing dyslipidemias.

**Table 2:** Absolute and relative medical contraindications to KMT.

**Health condition**	**Cervenka 2021^(35)^**	**Watanabe 2020^(36)^**	**Kirkpatrick 2019^(34)^**
**Absolute contraindications (according to one or more sources)**			
Breastfeeding		✓	
Cancers: kidney cancer, melanoma		✓	
Diabetes type I without endocrinology approval and/or supervision	✓	✓	
Heart failure (New York Heart Association class IV)		✓	RC
Hypercholesterolemia, severe inherited forms	RC	RC	✓
Hypertriglyceridemia, severe	RC	RC	✓
Infections (active/severe)		✓	
Liver failure	✓	✓	✓
Pancreatitis history	RC	RC	✓
Pancreatitis, acute	✓		✓
Porphyria	✓	✓	
Pregnancy	NC	✓	
Rare inborn errors of metabolism affecting fatty acid transport/oxidation including organic acidurias and primary carnitine deficiency^*^	✓	✓	
SGLT-2 inhibitor use (risk of euglycemic ketoacidosis)		✓	✓
Surgery/invasive procedures: discontinue KD 48 h prior		✓	
**Relative contraindications**			
Anorexia nervosa	✓		
Antihypertensive medication use	✓		✓
Cachexia	✓		
Cardiovascular disease	✓	✓	✓
Carnitine deficiency, secondary	✓		
Cerebrovascular disease	✓	✓	
Cholecystectomy	✓		
Diabetes medication use	✓	✓	✓
Diabetes type I (with endocrinology approval and/or supervision)	✓	✓	✓
Diabetes type II (with endocrinology approval and/or supervision)	✓		✓
Frail elderly		✓	
Gastroesophageal reflux, severe	✓		
Hyperlipidemias, any type^**^	✓	✓	^**^
Hyperuricemia		✓	
Kidney disease, mild to moderate	✓	S	✓
Kidney failure, advanced kidney disease	NC	✓	✓
Liver disease, mild to moderate		✓	✓
Mental disorders or substance abuse		✓	
Nephrolithiasis (past or present)	✓		
Osteopenia/osteoporosis	✓		
Respiratory failure		✓	
Social/behavioral factors: inadequate support, poor adherence, difficulty maintaining adequate nutrition	✓	✓	
Vitamin K-dependent anticoagulation therapy (due to potential increase in vitamin K intake from certain vegetables on KD)			✓

Blank = not addressed/no position; NC = no consensus reached; RC = relative contraindication; S = considered safe, ✓ = viewed as a contraindication by this source. ^*^See Cervenka et al. for complete list. ^**^Kirkpatrick et al. consider severe hyperlipidemias to be absolute contraindications and all other forms of hyperlipidemia to be relative contraindications.

Where relative contraindications exist, skilled medical supervision or clinical guidance is required, for example during breastfeeding (41) and in type I and type II diabetes (42).

Although documented instances of clinically concerning psychiatric symptoms emerging, resurfacing, or worsening in the context of KMT implementation are rare, this may be due to the limited number of published cases and clinical trials in this young field to date. Given that it can take several weeks to several months for mental health benefits of KMT to manifest, the authors recommend that KMT not be used as a standalone intervention for acute, unstable, or potentially dangerous psychiatric presentations such as new or worsening mania, new or worsening psychosis, active suicidal ideation, self-injurious behavior, severe agitation, aggressive behavior, intoxication, or mental status changes. The absence of published clinical experience in cases such as these is yet another reason to caution against the use of KMT as a primary intervention. Even when considering a trial of KMT to help manage serious symptoms that have already been safely stabilized, additional safeguards may be required such as specialist consultation, medication support, intensive outpatient treatment, residential care, or inpatient hospitalization.

Comorbid psychiatric conditions such as substance use disorders (e.g., alcohol and other drug use disorders) and advanced cognitive impairment should also be taken into consideration during treatment planning. Finally, although KMT is being explored as a potentially therapeutic adjunctive tool in the management of residual eating-disordered thoughts and behaviors in people with *weight-recovered* anorexia nervosa (43), the authors currently view *underweight* anorexia nervosa as an absolute contraindication to KMT.

Respondents agreed that, outside of an absolute contraindication, KMT should be considered as an adjunct therapy to first-line treatments such as psychotropic medications and psychotherapy for all individuals with serious mood and psychotic illnesses (S8, 98%), including:

1. Individuals with treatment-refractory serious mood and/or psychotic illness (S14, 98%)
2. Individuals who do not tolerate pharmaceutical treatments (S12, 94%)
3. Individuals who do not wish to use pharmaceutical treatments (S13, 98%)
4. Individuals with peripheral signs of metabolic dysfunction (S6, 91%)
5. Individuals who wish to undertake a trial of KMT (S16, 98%)

The likelihood of success of KMT increases when support is in place (S10, 89%). Individuals with less motivation or support may benefit from an intensive outpatient, residential, or inpatient care setting (S11, 100%).

KMT (by definition) is intended to address metabolic dysfunction and is therefore recommended for individuals who exhibit one or more signs of metabolic dysfunction (S6, 91%). These include elevations in fasting glucose, hemoglobin A1c, fasting insulin, triglycerides, triglyceride-to-HDL ratio, blood pressure, and waist-to-height ratio (a measure of central obesity). Even when observable signs of peripheral metabolic dysfunction are absent, KMT is still worth considering, as metabolic dysfunction may still be present in the central nervous system (S7, 89%) (44, 45).

In cases where KMT is not appropriate, feasible, or desired, personalized nutrition counseling focused on improving overall dietary quality may bring metabolic and mental health benefits and is therefore well worth considering; however, an evaluation of specific dietary strategies other than ketogenic dietary therapy was not conducted by the steering group as it was beyond the scope of this endeavor.

#### 4.1.3 Topic C: The monitoring and measurement standards for ketogenic therapy

The authors recommend that initial assessment include a complete blood count (CBC), comprehensive metabolic panel (CMP), fasting lipid profile, fasting insulin, vitamin B12, vitamin D3, and a carnitine panel (S23, 94%). They further recommend that these parameters be monitored regularly during KMT (S24, 100%) and that any nutrient deficiencies be addressed with supplementation (S25, 98%). Additional testing should be tailored according to each specific case.

The steering group did not establish consensus views on the specifics of carnitine assessment and replacement in the management of serious mental illness, as experience and evidence in this area is limited. However, guidelines for the management of adults with epilepsy support supplementation if levels are low or if symptoms associated with carnitine deficiency are present (35). Carnitine is a conditionally essential nutrient responsible for transporting long-chain fatty acids into mitochondria in preparation for oxidation. If endogenous synthesis (from lysine and methionine) or exogenous supply (from dietary sources) of carnitine is insufficient to meet this increased demand, fat metabolism and ketone production could be compromised, resulting in lower than expected BHB levels and potentially also indicators of energy deficit, such as fatigue, hypoglycemia, or cognitive difficulties. Indeed, reductions in carnitine levels are not uncommon in adults following a KD for the management of epilepsy, and lower levels may be associated with poorer seizure control (46).

Risk factors for acquired carnitine deficiency that make close monitoring especially important include use of valproate, liver disease, kidney disease, diets low in carnitine, lysine, and/or methionine (including vegan and vegetarian diets), malnutrition, type II diabetes, advanced age, and use of cyclosporin or pivampicillin (47–49). If free plasma carnitine is below 25 μmol/L, acetyl-L-carnitine in divided doses of up to 100 mg/kg per day as tolerated may be useful. As carnitine bioavailability from supplements is far lower than from foods, dietary counseling may also be worthwhile.

Broadly, the aim of KMT is to maintain ketosis within a range that achieves symptom management (35). Respondents agreed that clinically relevant ketosis requires blood BHB concentrations of at least 0.5 mmol/L (S17, 100%), and that for some patients, the greatest improvements in psychiatric symptoms occur when BHB levels are maintained at ≥1.0 mmol/L (S18, 96%). To manage ketosis effectively, the authors recommend monitoring ketone levels frequently—at least once per day—during the initiation phase (up to three months), and periodically as needed thereafter. Because ketone levels can vary substantially throughout the day, taking measurements at approximately the same time each day may make it easier to identify trends and interpret results over time. While the authors recommend blood testing of BHB as the gold standard for accuracy (50), urine or breath monitoring should be considered in cases where blood monitoring is not an option (S22, 96%). Continuous glucose monitoring (e.g., for patients prescribed glucose-lowering medications) and continuous ketone monitoring (where available) could be useful in select clinical contexts and may therefore be considered by the treating medical team on a case-by-case basis.

#### 4.1.4 Topic D: The best practices in employing ketogenic therapy

The safe, appropriate implementation of KMT in serious mood and psychotic illnesses ideally involves a multidisciplinary team of healthcare professionals (51), including, at a minimum, a nutrition professional and a prescribing clinician with training and experience in KMT (S28, 91%). In some cases, these roles may be combined if the prescribing clinician is also trained in nutrition and experienced in administering KMT. The team may also include a therapist, nurse, and/or health coach. All team members should be suitably educated regarding the safe and appropriate use of KMT in mental health conditions, but the standard of “expert KMT practitioner” has yet to be defined, therefore further work should be undertaken to establish training criteria (S33, 96%).

Due to metabolic variations between patients, there is no “one-size-fits-all” dietary protocol that will result in sustainable therapeutic ketosis for everyone, therefore KMT protocols must be tailored to the individual. The classic ketogenic diet first used in the management of pediatric epilepsy over a century ago employed a macronutrient ratio of 4:1 (4 grams of fat to every 1 gram of protein plus carbohydrate). Over time it has been observed that seizure control can be achieved in many cases in both children and adults using modified versions of the diet that allow for more protein and/or carbohydrate, therefore a number of less restrictive variations on the original protocol now exist to choose from. These include modified ketogenic diets, modified Atkins diets, and low-glycemic index diets (35, 52, 53). While the efficacy of less restrictive ketogenic protocols is well documented in epilepsy management, similar evidence has not yet been established for the treatment of serious mental illness.

Dietary design and personalization should be managed by a registered dietitian, licensed nutritionist, prescribing clinician, or health coach trained and experienced in KMT implementation (S28, 91%). Adequate hydration and electrolyte intake are important during the initiation and maintenance of KMT (S26, 98%).

Any medications and pre-existing health conditions must be skillfully and attentively managed by the prescribing clinician(s) on the team, as physiological adaptations to ketosis can affect blood pressure, blood glucose levels, electrolyte balance, fluid status, medication levels, and medication effects in ways that may require more frequent clinical evaluation, laboratory monitoring, and medication adjustments (S28, 91%; S29, 96%; S31, 100%; S32, 98%) (54–58).

During early adaptation to KMT, in addition to the physiological symptoms described above, it is possible that psychiatric symptoms may emerge or worsen. These may include depression, difficulty concentrating, fatigue, hypomania, mania, insomnia, anxiety, irritability, mood swings, or psychosis. Although these usually abate within a few weeks or less, it is important that patients and caregivers are made aware of this possibility, educated to recognize any worsening of symptoms, and advised to immediately notify the healthcare team should this occur (S30, 91%). Clinical assessment including vital signs and laboratory tests is warranted to look for contributing factors associated with keto-adaptation including inadequate electrolyte support, dehydration, hypotension, hypoglycemia, hyperketonemia, mild metabolic acidosis, and/or diet-drug interactions that may require adjustments to the treatment plan. Symptoms commonly associated with keto-induction such as headache, lightheadedness, fatigue, decreased exercise capacity, mood changes, muscle cramps, and gastrointestinal disturbances may be less likely to occur with dietary protocols that allow for gradual rather than abrupt transition into ketosis (58).

Should any symptoms arise that pose safety concerns, fail to respond to mitigation strategies, or become too distressing or uncomfortable to tolerate, KMT can be readily discontinued by raising carbohydrate intake to at least 100 g per day.

There is no evidence to suggest how long KMT may need to be continued once mental health treatment goals have been achieved. However, as serious mood and psychotic illnesses are often long-term conditions, individuals may wish to remain on KMT on a long-term basis to maintain mental health benefits, especially given that experience using KMT in epilepsy suggests that seizures can recur when KMT is discontinued (35). Given that adherence to ketogenic diets can be challenging, KMT treatment planning should ideally include the development of a personalized contingency plan (which may involve a higher level of support and/or temporary use of medication) should ketosis be interrupted and serious psychiatric symptoms re-emerge. Further evidence and collective clinical experience using KMT in serious mood and psychotic illnesses are required to provide specific guidance regarding duration of treatment and best practices for safe discontinuation.

### 4.2 Strengths and limitations

The agreement level achieved for all statements was ≥89%—an unanticipated level of agreement for an anonymous survey concerning an area of medicine currently unsupported by randomized control trial evidence. This may indicate that the statements were designed to be agreeable (consensus for which, therefore, adds little value), or that the panel of respondents for Round 2 was selected to demonstrate alignment with the steering group. The use of a 4-point Likert scale helped force responders to form an opinion and avoid middle option bias. However, it is acknowledged this may have introduced a greater acquiescence bias effect. In addition, it should be acknowledged that there are different levels of agreement/disagreement and “tend to agree/disagree” responses should be considered to be weaker than “strongly agree/disagree”. Although there is potential bias in the steering group recruitment of the responder panel, it should be noted that as a nascent area of medicine, the community of healthcare professionals working with KMT in psychiatric conditions is limited, and there is little value in gathering opinions from those who have little or no awareness of this treatment modality in practice. It is perhaps reassuring that agreement levels were so high, as this validates the statements devised by the steering group, and allows the steering group to establish a set of practical recommendations intended to support healthcare professionals using KMT in serious mental illness.

A notable limitation is the lack of patient perspectives and experience regarding KMT. which may have a significant bearing on the practicability and implementation of KMT in serious mood and psychotic illnesses.

### 4.3 Recommendations

Based on the modified Delphi findings, the authors propose the following recommendations for implementation of KMT in adults with major depressive disorder, bipolar disorder, and schizophrenia.

1 KMT should be considered as adjunct therapy to first-line treatments for major depressive disorder, bipolar disorder, and schizophrenia.
2 Individuals with major depressive disorder, bipolar disorder, or schizophrenia who decline, cannot access, do not tolerate, or have not sufficiently benefited from first-line treatments should also be considered for a trial of KMT if they qualify.
3 The absence of observable signs of metabolic dysfunction should not preclude individuals with schizophrenia, bipolar disorder, or major depressive disorder from a trial of KMT.
4 At a minimum, the healthcare team should ideally include a nutrition professional and a prescribing clinician who have been trained in the safe implementation of KMT.
5 Prior to consideration of KMT, a thorough medical and psychiatric history should be taken to assess for the presence of absolute and relative contraindications.
6 Prior to initiating KMT, a baseline assessment that includes a CBC, CMP, fasting lipid profile, vitamin D level, vitamin B12 level, and carnitine panel is recommended.
7 The approach to achieving ketosis should be well formulated and tailored to the needs and preferences of the individual, with consideration for the long-term sustainability of dietary modifications.
8 The goal of KMT is to achieve and sustain a degree of ketosis that could potentially lead to improvements in serious mood and/or psychosis symptoms. Ketones should therefore be monitored regularly during KMT, preferably by measuring capillary blood levels of BHB.
9 While a BHB concentration of 0.5 mmol/L indicates a state of ketosis, many patients will require levels to be consistently maintained at 1.0 mmol/L or higher for best outcomes.
10 Barring any medical or psychiatric eventualities that may necessitate earlier discontinuation, for a trial of KMT to be considered adequate, a state of ketosis should be maintained for at least three consecutive months before drawing conclusions about its effectiveness.

### 4.4 Conclusion

This modified Delphi exercise was able to achieve consensus across a panel of 47 healthcare professionals and identify recommendations for the safe and efficacious use of KMT in the treatment of major depressive disorder, bipolar disorder, and schizophrenia. As clinical experience and research in this area grows, it is intended that this study will be repeated in 5 years (potentially including patient perspectives) to understand how expert opinions may have shifted.

## Data Availability

The original contributions presented in the study are included in the article. Further inquiries can be directed to the corresponding author.
